# DC-SIGN Increases the Affinity of HIV-1 Envelope Glycoprotein Interaction with CD4

**DOI:** 10.1371/journal.pone.0028307

**Published:** 2011-12-07

**Authors:** Karolin Hijazi, Yufei Wang, Carlo Scala, Simon Jeffs, Colin Longstaff, Daniel Stieh, Beth Haggarty, Guido Vanham, Dominique Schols, Jan Balzarini, Ian M. Jones, James Hoxie, Robin Shattock, Charles G. Kelly

**Affiliations:** 1 King's College London, Dental Institute, Oral Immunology, Tower Wing, Guy's Hospital, London, United Kingdom; 2 Jefferiss Trust Research Laboratories, Wright-Fleming Institute, Division of Medicine, Faculty of Medicine, Imperial College, London, United Kingdom; 3 Biotherapeutics Section, National Institute for Biological Standards and Control, Potters Bar, Hertfordshire, United Kingdom; 4 Centre for Infection, Department of Cellular and Molecular Medicine, St George's, University of London, London, United Kingdom; 5 Penn Center for AIDS Research, University of Pennsylvania, Philadelphia, Pennsylvania, United States of America; 6 Virology Unit, Division of Microbiology, Department of Biomedical Sciences, Institute of Tropical Medicine, Antwerp, Belgium; 7 Department of Biomedical Sciences, University of Antwerp, Antwerp, Belgium; 8 Faculty of Medicine and Pharmacy Free University of Brussels, Brussels, Belgium; 9 Rega Institute for Medical Research, Katholieke Universiteit Leuven, Leuven, Belgium; 10 School of Biological Sciences, University of Reading, Reading, United Kingdom; Academic Medical Center, Netherlands

## Abstract

Mannose-binding C-type lectin receptors, expressed on Langerhans cells and subepithelial dendritic cells (DCs) of cervico-vaginal tissues, play an important role in HIV-1 capture and subsequent dissemination to lymph nodes. DC-SIGN has been implicated in both productive infection of DCs and the DC-mediated *trans* infection of CD4^+^ T cells that occurs in the absence of replication. However, the molecular events that underlie this efficient transmission have not been fully defined. In this study, we have examined the effect of the extracellular domains of DC-SIGN and Langerin on the stability of the interaction of the HIV-1 envelope glycoprotein with CD4 and also on replication in permissive cells. Surface plasmon resonance analysis showed that DC-SIGN increases the binding affinity of trimeric gp140 envelope glycoproteins to CD4. In contrast, Langerin had no effect on the stability of the gp140:CD4 complex. *In vitro* infection experiments to compare DC-SIGN enhancement of CD4-dependent and CD4-independent strains demonstrated significantly lower enhancement of the CD4-independent strain. In addition DC-SIGN increased the relative rate of infection of the CD4-dependent strain but had no effect on the CD4-independent strain. DC-SIGN binding to the HIV envelope protein effectively increases exposure of the CD4 binding site, which in turn contributes to enhancement of infection.

## Introduction

Dendritic cell (DC) subsets [1]–[3] as well as Langerhans cells (LCs) [4]–[6] in genital mucosal tissue may play a key role in transmission of human immunodeficiency virus type 1 (HIV-1) to CD4^+^ T cells. While CD4^+^ T cells form the founder populations of infected cells at the portal of entry [5], [7], DCs and LCs contribute to viral dissemination to lymphoid tissues and enhance amplification of viral replication in CD4^+^ T cells at mucosal sites [8]. DCs and LCs bind HIV and transfer virus to permissive CD4^+^ T cells in a process termed *trans* infection that does not require HIV replication in DCs or LCs [4], [9]. In addition, immature DCs and LCs express low levels of cell surface CD4 and CCR5 and are susceptible to infection with HIV [10]–[12]. Although replicative infection in DCs and LCs is much less efficient than in CD4^+^ T cells and macrophages [5], [13], [14], infected DCs and LCs can efficiently release *de novo* synthesized virus particles to CD4^+^ T cells at the points of cell contact termed virological synapses [5], [15]–[17]. Thus DC-mediated transmission of virus involves two different mechanisms that can be distinguished temporally [17]. Within 24 hours of exposure to HIV, DCs transmit either surface bound virus or internalised virus *in trans* (in the absence of productive replication) [18]. Beyond this time-point, immature DCs that have been infected transmit progeny rather than input virus to permissive target cells that express CD4 and chemokine receptors [16], [17].

Mannose-binding C-type lectin receptors expressed on the surface of LCs and subepithelial DCs of cervico-vaginal tissues bind the highly glycosylated HIV envelope protein and capture HIV [9], [11], [19], although other unidentified receptors may also bind HIV [20]. In particular, the C-type lectin DC-SIGN (DC specific ICAM-3-grabbing nonintegrin) has been identified as a cell surface receptor on immature DCs that binds HIV and mediates transfer of virus to CD4^+^ permissive T cells [9], [15], [18], [21], [22]. DC-SIGN binding to HIV results in internalisation of virus to a non-endolysosomal compartment [17], [18]. From this compartment, internalised virus moves rapidly to synapses formed by infected DCs and CD4^+^ T cells, however, within 24 hours HIV in this compartment is degraded concomitant with a decline in transfer of infectious input virus [17].

DC-SIGN binding to HIV may also enhance DC infection directly and so contribute to the second longer-term mechanism of DC-mediated infection that involves transfer of progeny virus to CD4^+^ T cells [17], [23]. Co-expression of DC-SIGN with CD4 and CCR5 in transfected cell lines or in T cell lines resulted in modest (two- to five-fold) increases in infection with HIV-1 although the relative enhancement was increased in cell lines that expressed lower levels of CCR5 [22], [24]. DC-SIGN binding to HIV-1 anchors the virus and may provide an increased local concentration of virus at the DC surface that facilitates interaction with CD4 and co-receptor [22], [24].

Not all mannose-binding C-type lectin receptors enhance infection either *in trans* or when expressed *in cis* with CD4 and co-receptor. In contrast to DC-SIGN, the LC-specific lectin Langerin mediates a protective effect since binding of HIV results in internalisation into Birbeck granules and rapid degradation [25]. Thus binding of HIV at the cell surface is not sufficient *per se* to enhance infection.

In this study, we have investigated the interaction between DC-SIGN or Langerin with gp140 (soluble, trimeric ectodomain of HIV envelope glycoprotein) to determine whether factors other than concentration at the cell surface also contribute to *cis* enhancement of infection. Surface plasmon resonance assays demonstrate that binding of DC-SIGN, but not Langerin, to HIV gp140 considerably increases the affinity of binding of gp140 to CD4. Enhancement of infection *in vitro* of permissive cells that express DC-SIGN was greater for the CD4-dependent HIV-1 IIIB strain than for the CD4-independent strain HIV-1 IIIBx [26]. This is consistent with the proposal that DC-SIGN may promote infection of immature dendritic cells by both concentrating virus at the cell surface and promoting binding to CD4.

## Materials and Methods

### Reagents and cells

The human anti-gp41 Mab 5F3 [27] was a kind gift of D. Katinger (Polymun GmbH, Vienna, Austria). The mouse anti-DC-SIGN Mab 120507 was from R&D Systems (Abingdon, Oxon, UK). CHO-expressed soluble CD4, Mab b12 [28] and Mab 447-52D [29], [30] were obtained through the Centralised Facility for AIDS Reagents (CFAR), National Institute for Biological Standards and Controls (NIBSC, Potters Bar, Herts, United Kingdom) and were donated by Progenics Pharmaceuticals, Inc., USA, D. P. Burton and S. Zolla-Pazner, respectively. The lectin HHA was prepared as described [31]. T-20 (Fuzeon) was from Roche, Welwyn Garden City, UK. Mannan was purchased from Sigma-Aldrich (Poole, UK). Recombinant *E. coli* DnaJ was provided by E. McGowan (King's College London, London, UK).

### Cells

PM1 cells (CFAR, NIBSC, donated by Paolo Lusso) [32], THP-1_ATCC_ and THP-1_ATCC_/DC-SIGN cells (referred to as THP-1 and THP-1DC-SIGN hereafter) (NIH AIDS Research and Reference Reagent Program, Division of AIDS, NIAID, NIH, Germantown, MD, USA, original source L. Wu and V. N. KewalRamani) [33] were grown in RPMI 1640 medium supplemented with 10% foetal calf serum, 100 U/ml penicillin, 100 µg/ml streptomycin and 2 mM L-glutamine. 293T/17 cells (ATCC #CRL-11268, Manassas, VA, USA), used for expression of recombinant gp140, were maintained in Dulbecco's Modified Eagles Medium (DMEM) (Invitrogen, Paisley, UK) supplemented with 10% foetal calf serum. Cells were grown in an environment enriched with CO_2_ (5%) at 37°C and passaged every 2–3 days or at approximately 80–90% confluence and the number of passages did not exceed 5. Immature monocyte-derived DCs were generated as previously described [21].

### Virus stocks

HIV-1 clade B strains 92FR_BX08 (BX08) [34], [35] and IIIB [36] were obtained through the CFAR, NIBSC and were donated by V. Polonis and R. Gallo, respectively. IIIBx, the CD4-independent variant of IIIB, was derived as previously described [37]. Viruses were propagated in peripheral blood mononuclear cells (PBMCs) isolated from buffy coats (National Blood Transfusion Service, London, UK) using a Ficoll-Hypaque density gradient. Prior to infection PBMCs were activated with phytohemagglutinin (PHA; 0.5 µg/ml; Sigma) and IL-2 (20 U/ml; Sigma). 50% tissue culture infective dose (TCID_50_) values of cell free viral stocks were determined in PM1 or THP-1 cells as previously described [38]. Infectivity was estimated by measurement of p24 antigen release in the supernatant by enzyme-linked immunosorbent assay (HIV-1 p24 Antigen ELISA Kit, Zeptometrix Corp, Buffalo, NY, USA) according to the manufacturer's instructions.

### Expression and purification of DC-SIGN

Complementary DNA of immature monocyte-derived dendritic cells was used as template to generate the DNA fragment encoding the entire extracellular domain of DC-SIGN (residues 70-404, GenBank accession number NP_066978) by PCR with primers 5′-GTCTCGAGATGGAACAATCCAGGCAAGACGCGATCT-3′ (sense) and 5′- TCGGATCCCTACGCAGGAGGGGGGTTTGGGGT-3′ (antisense). The amplified sequence, digested with *Xho*I and *Bam*HI was inserted in pET15b (Novagen, EMD Chemicals, Gibbstown, NJ, USA) and cloned in *E. coli* TOP10 (Invitrogen). Cloned fragments were confirmed by DNA sequencing (Advanced Biotechnology Centre, Imperial College London, London, UK) and compared with GenBank (accession number NM_021155). For expression, *E. coli* strain BL21/DE3 (Stratagene, La Jolla, CA, USA) was transformed with recombinant plasmid. Protein expression and refolding was performed as described [39] with minor modifications. Inclusion bodies (from 1 l bacterial culture) were recovered by centrifugation at 10,000× *g* for 20 min at 4°C, and solubilized in 8 ml of 100 mM Tris-HCl, pH 8.0 containing 6 M urea (solubilizing buffer) supplemented with 0.01% 2-mercaptoethanol, by gentle rotation for 16 h at 4°C. The mixture was centrifuged at 20,000× *g* for 30 min at 4°C and soluble recombinant protein was isolated by Ni^2+^ affinity chromatography. Bound material (recovered by elution with 200 mM imidazole in solubilizing buffer) was dialyzed against 2 l of 100 mM Tris-HCl, pH 8.0, 0.01% 2-mercaptoethanol, 10 mM CaCl_2_, 4 M urea then successively against the same buffer with 2 M urea and no urea. Final dialysis was against 100 mM Tris-HCl, pH 8.0, 10 mM CaCl_2_. After dialysis, insoluble precipitate was removed by centrifugation at 100,000× *g* for 30 min at 4°C and refolded DC-SIGN present in the soluble fraction was purified by D-mannose affinity chromatography as previously described [40]. Fractions were analyzed by SDS-PAGE and protein concentrations were determined by densitometric analysis using the GeneSnap software (Syngene, Cambridge, UK). The identity of the protein was confirmed by liquid chromatography tandem mass spectrometry (LC MS/MS) analysis (MRC Clinical Sciences Centre, Imperial College London, London, UK).

### Expression and purification of Langerin

The sequence encoding the full extracellular domain of Langerin (residues 56–328, Genbank accession number CAB62403) was synthesized and cloned (Epoch Biolabs, Missouri City, TX, USA) in pET15b ( Novagen). *E. coli* strain BL21/DE3 was transformed for expression. Inclusion bodies were recovered and protein purification and refolding was carried out as previously described [41]. The identity of the protein was confirmed by LC MS/MS analysis (MRC Clinical Sciences Centre, Imperial College London).

### Size exclusion chromatography

Refolded proteins (approximately 50 µg) were resolved by size exclusion chromatography on two tandemly connected Superdex 200 3.2/30 PC columns with the ÄKTAbasic system (GE Healthcare, Uppsala, Sweden) in 100 mM Tris-HCl, pH 7.9, 0.15 M NaCl with flow rate of 0.05 ml/min at room temperature. Absorbance was monitored at 280 nm, and fractions were analyzed by SDS-PAGE. Molecular mass was estimated by comparing the elution positions on the chromatogram to those of marker proteins injected under identical conditions (apoferritin, 400,000; α-amylase, 200,000; alcohol dehydrogenase, 150,000; transferrin, 80,000; bovine serum albumin, 67,000; ovalbumin, 45,000; β-lactoglobulin dimer, 36,000; carbonic anydrase, 30,000; myoglobin, 18,000).

### Expression and purification of gp140

Gene fragments encoding BX08 gp140 (residues 31–663) and IIIB gp140 (residues 31–665) were amplified from pSFVBX08wtgp160 (kind gift of Ralf Wagner, University of Regensburg, Germany), harbouring the gp160 sequence derived from BX08, and the molecular clone pBH10 (CFAR, NIBSC), respectively, modified and inserted into the pEE14/tpa vector (Lonza Biologics plc, Slough, UK) under control of the human tissue plasminogen activator signal peptide as previously described for production of envelope glycoproteins from six primary isolates of HIV [42], [43]. For expression, the recombinant pEE14 plasmids were transfected into 293T/17 cells using the transfection reagent Polyethyleminine “MAX” (12.5 µg/ml, PEI MAX, Polysciences Inc., Warrington, PA, USA) as described [44]. Cells were incubated at 32°C for 4 h with the DNA-PEI complex, washed with 100 ml PBS and then incubated in DMEM+0.05% foetal calf serum at 32°C for a maximum of 72 h after which cell culture supernatants were harvested for purification.

Recombinant gp140 was purified by GNA affinity chromatography as described followed by size-exclusion chromatography on a Superdex G200 column (GE Healthcare) to enrich for trimeric gp140 [42]. Purified fractions corresponding to the major peaks were analyzed by SDS-PAGE and immunoblotting and protein concentrations were determined by optical density measurement at 280 nm.

### Surface plasmon resonance assays

For protein immobilization by direct amine coupling to the surface of CM5 sensorchips (GE Healthcare), samples were dissolved in 10 mM Na acetate (pH 4.0). For binding studies, HBS-P buffer (10 mM HEPES [pH 7.4], 0.15 M NaCl, 0.005% vol/vol surfactant P20; GE Healthcare) supplemented with 10 mM CaCl_2_, was used. Flow rate was 20 µl/min. Equilibrium dissociation constants (K_D_) as well as association (k_a_) and dissociation constant (k_d_) rates were calculated using the BIAevaluation software 4.1 (GE Healthcare). Curves were fitted to the model which gave the best fit as judged by the lowest Chi^2^ value and best distribution of deviation from the calculated fit.

#### Determination of gp140 direct binding to DC-SIGN and Langerin

Gp140 at 0.5 µg/ml was immobilized on the sensorchip surface of flow cell 2 by direct amine coupling (560 and 620 resonance units (RU) for BX08 or IIIB, respectively). Flow cell 1 served as reference. Binding of fluid phase DC-SIGN (50–600 nM) or Langerin (25–200 nM) was then determined. The sensorchip surface was regenerated with 10 mM EDTA.

#### Effect of soluble DC-SIGN and Langerin on stability of gp140 interaction with CD4

Mab 5F3 (2 µg/ml) was immobilized by direct amine coupling on both flow cell 1 and flow cell 2 (approximately 6000 and 5500 RU, respectively) and gp140 (100 nM) was injected over flow cell 2. DC-SIGN or Langerin (both at 50 nM) were then injected over both flow cells followed by injection of gp140 ligands (CD4, Mab b12, or Mab 447-52D) at varying concentrations. Reproducibility of binding of gp140, DC-SIGN or Langerin binding was verified by repeated injections. Difference sensorgrams of CD4 binding were obtained after subtracting dissociation of 5F3:gp140 or 5F3:gp140:DC-SIGN/Langerin complexes. Because of the very low dissociation rate of the gp140:CD4 complex in the presence of DC-SIGN, rate constants could not be calculated for this binding. To allow comparison between binding ± DC-SIGN, values for the half-life (t_1/2_) of the complexes were calculated using the formula t_1/2_ = ln 2/k_d_ (t_1/2_ = 0.693/k_d_).

No binding of DC-SIGN or CD4 to immobilised 5F3 was evident in the control flow cell. In addition, in separate experiments where CD4 was injected over immobilised DC-SIGN, no binding of CD4 was detected.

### Flow cytometry

To assess binding of soluble DC-SIGN to PM1 cells, serial dilutions of protein (5–350,000 pM) were incubated with PM1 cells (0.25×10^6^) for 30 min at 4°C. Cells were washed three times with cold PBS and incubated with 5 µl of anti-DC-SIGN Mab (10 µg/ml, clone 120507) for 30 min followed by a further 30 min incubation with FITC-conjugated goat anti-mouse IgG (Dako, Cambridgeshire, UK). An isotype control murine antibody of irrelevant specificity was used as negative control. Binding of HHA to PM1 cells was assessed by incubation of PM1 cells with serial dilutions of FITC-conjugated HHA (EY Laboratories, San Mateo, CA, USA) for 30 min at 4°C. After incubation, cells were extensively washed with PBS. FITC-conjugated *Lotus tetragonolobus* lectin (Vectorlabs, Peterborough, UK) was used as negative control. For some experiments, mannan (100 µg/ml) was added to DC-SIGN or HHA before incubation with cells. The cells were then analysed by a BD FACSCanto II flow cytometer using the FACSDiva software (Becton Dickinson, Oxford, UK) and data analysis was performed using the WinMDI 2.9 software.

### Purification of viral particles and virus binding assays

Viral particles were inactivated using aldrithiol-2 as previously described [45]. Inactivated virus was concentrated by spinning on a 17–25% sucrose cushion at 35,000 rpm in a SW55Ti centrifuge for 16 h as previously described [46]. Pellets were resuspended in PBS supplemented with 10 mM EDTA and 1% BSA. CD45 microbeads (Miltenyi Biotec, Bergisch Gladbach, Germany) were added at a concentration of 10 µl/µg p24 and incubated with mixing for 4 h. Virus was then concentrated again by centrifugation on a 25% sucrose cushion at 55,000 rpm for 1 h. Samples were then resuspended in degassed PBS with 1 mM EDTA for binding experiments. Binding activity of virions to soluble DC-SIGN was assessed on a RapID4 acoustic biosensor (TTP Labtech). Soluble DC-SIGN and BSA were covalently bound on the experimental and control flow cells respectively by direct amine coupling. Serial dilutions of purified or unpurified virus (34–250 nM, determined by p24 ELISA and normalized assuming 2500 gag proteins per virion [46]) were prepared and allowed to bind to the surface for 3 min, followed by 5–10 min of dissociation. For experiments with unpurified virus, cell culture supernatant of uninfected cells was used as negative control. Surfaces were regenerated with 100 mM glycine-HCl, pH 2.5. Data were fitted to the Langmuir kinetic model.

### Cell viability assays

Cell viability was assessed by the *in situ* reduction of 3-(4,5-dimethylthiazol-2-yl)-2,5-diphenyltetrazolium bromide (MTT assay) [47]. PM1 cells (0.2×10^6^) were seeded in 96-well plates and exposed to recombinant proteins for 72 h at 37°C. Cells were washed with PBS and treated with MTT (0.5 mg/ml; Sigma) in RPMI for 3 h. Cells were then solubilized overnight with 20% SDS in H_2_O∶DMF (1∶1) and the absorbance determined at 570 nm, with reference at 630 nm. Viability was estimated by comparison with viable cells exposed to medium alone and assays were performed in triplicate.

### Infections

For *cis*-enhancement assays, infection of the permissive cell line THP-1 was compared with that of THP-1DC-SIGN. THP-1 and THP-1DC-SIGN cells were infected with HIV IIIB or HIV IIIBx at MOI of 1–4×10^−4^. Two hours post-infection unbound virus was washed off with three volumes of RPMI. After 10 days, p24 levels were measured by HIV p24 antigen ELISA. For assessment of viral binding, THP-1 and THP-1DC-SIGN cells were incubated with HIV IIIB or HIV IIIBx at p24 concentrations of 0–100 ng/ml. After 2 h cells were lysed with 1% Triton-X and p24 levels were measured by ELISA. For infection assays with soluble lectins, serial dilutions (in triplicate) of test protein (0–40,000 pM) were pre-incubated with HIV-1 (BX08 or IIIB) at 10^−4^ MOI (as determined in PM1 cells) for 30 min at 37°C and subsequently added to PM1 cells (0.4×10^5^) in 96-well, round bottom, tissue culture plates. For some experiments, mannan (10 µg/ml) was added to the mixture of DC-SIGN and virus. Cultures were incubated at 37°C for 10 days. Viral replication was measured by p24 ELISA and defined as %p24 protein release in the absence of test compound, which was defined as 100% and corresponded to 68–100 pg/ml. 50% inhibitory concentrations (IC_50_) were estimated by linear regression analysis using GraphPad Prism 5 software (San Diego, CA). For all experiments heat-inactivated virus (1 h at 56°C) was used as background correction. Each condition was assayed in a minimum of three independent experiments.

### Spinoculation and fusion kinetics assays

To measure the rate of virus-cell fusion we used spinoculation to provide synchronous infection [48], [49]. Viruses (IIIB or IIIBx) at 3×10^−4^ MOI were mixed with THP-1 and THP-1DC-SIGN cells and centrifuged at 1,200× g, 4°C for 3 hours. Cultures were then washed with fresh medium and warmed to 37°C prior to addition of T-20 or Mab b12 at 10 µg/ml at the following time points: 0, 15, 30, 60, 120, 180, 240 and 360 min. Levels of p24 in the supernatant were assayed after 4 days and relative infectivity values were defined as %p24 protein released in the absence of inhibitor for each virus and cell type, which was defined as 100%. Heat-inactivated virus (1 h at 56°C) was used as background correction. Each condition was assayed in triplicate in 4 independent experiments.

### Statistics

Analyses were performed using GraphPad Prism 5. Normal distribution of data was demonstrated using the D'Agostino-Pearson normality test. Comparison of two data sets and estimation of two-tailed P values was carried out using unpaired *t* test with Welch's correction.

## Results

### Expression and characterization of recombinant DC-SIGN and Langerin

**Figure 1:**
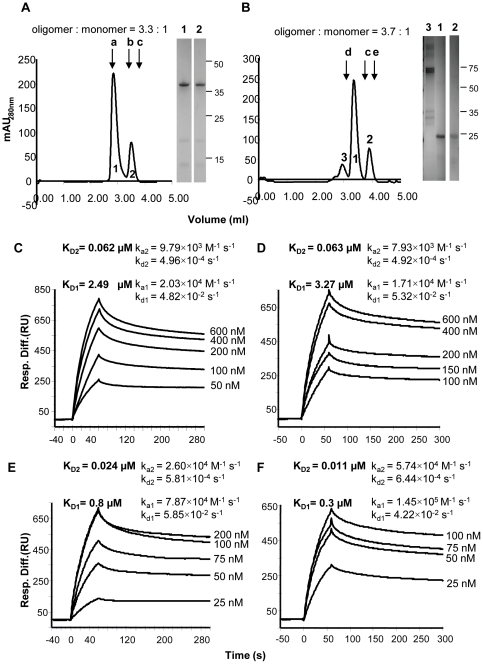
Oligomeric state and binding activity of soluble DC-SIGN and Langerin preparations. (**A, B**) Size exclusion chromatogaphy of DC-SIGN and Langerin, respectively. Elution positions of molecular mass standards (a: α-amylase, 200,000; b:ovalbumin, 45,000; c:carbonic anydrase, 30,000; d:transferrin, 76,000; e: myoglobin, 18,000) are arrowed. Inserts in panels show SDS-PAGE analyses of peak fractions as indicated. (**C, D**) Superimposed sensorgrams of fluid phase DC-SIGN , binding to immobilised BX08 gp140 or IIIB gp140, respectively. Concentrations of fluid-phase components are indicated. (**E, F**) Superimposed sensorgrams of fluid-phase Langerin, binding to immobilised BX08 gp140 or IIIB gp140, respectively. Kinetic constants, calculated by using the heterogeneous analyte model (BIAevaluation 4.1 software), are indicated. K_D2_ and K_D1_ are equilibrium binding constants of the oligomeric and monomeric, respectively, forms of the lectins.

Recombinant polypeptides comprising the extracellular domains of DC-SIGN and Langerin, respectively, were expressed in *E. coli*, refolded and purified by affinity chromatography on Ni^2+^ and mannose resins sequentially. Identity of the polypeptides was confirmed by peptide sequence analysis. Refolded DC-SIGN bound to Mab 120507 with K_D_ of approximately 2×10^−9^ M as determined by surface plasmon resonance (data not shown) in agreement with previous findings [50]. The oligomeric state of DC-SIGN and Langerin was determined by size exclusion chromatography. Both proteins eluted as two well-resolved major peaks corresponding to the molecular mass of the monomeric and tetrameric forms of DC-SIGN (Fig. 1A) [50], and the monomeric and trimeric forms of Langerin (Fig. 1B). Some non-lectin components were also evident in a minor peak (labeled 3) in the Langerin preparation. The relative distribution of the higher and the lower molecular mass forms was 3.3∶1 for DC-SIGN and 3.7∶1 for Langerin. When fractions from the higher molecular mass peak were re-chromatographed, the proteins redistributed with approximately the same proportion of higher and lower molecular mass forms (data not shown). In these preparations, the tetrameric form of DC-SIGN and the trimeric form of Langerin predominate in monomer∶oligomer equilibria.

#### Recombinant DC-SIGN and Langerin bind HIV-1 gp140

Binding of recombinant DC-SIGN and Langerin to soluble trimeric gp140 envelope glycoproteins was measured by surface plasmon resonance. For these experiments, gp140 from HIV-1 BX08 (R5 clade B) or IIIB (X4 clade B) was immobilized directly on the sensorchip surface and binding of fluid phase DC-SIGN or Langerin was determined over a range of concentrations (50–600 nM for DC-SIGN, 25–200 nM for Langerin). Superimposed sensorgrams are shown in Fig. 1C–F. Data could not be fitted to a 1∶1 Langmuir binding model using the BIAevaluation software but, for both DC-SIGN and Langerin, data fitted to a heterogeneous analyte model in which it is assumed that both lectin preparations include oligomeric and monomeric forms in the proportions indicated by the gel filtration analyses. The equilibrium dissociation constants (K_D2_) calculated for the tetrameric form of DC-SIGN were approximately two orders of magnitude lower than those calculated for the monomer (Fig. 1C and 1D). The difference can be attributed to the lower dissociation constant (k_d_) estimated for the tetramer compared with that of the monomer. In this model, tetrameric DC-SIGN is the dominant form of the lectin that binds to gp140. The K_D2_ values for tetrameric DC-SIGN binding to gp140 (BX08) and gp140 (IIIB) were approximately 0.062 µM and 0.063 µM, respectively, in agreement with values (0.003–0.061 µM) reported previously for DC-SIGN binding to gp120 from 4 different strains of HIV-1 [50]. The trimeric form of Langerin also bound to gp140 with higher affinity than the monomer again attributed to the slower dissociation of the trimer. Trimeric Langerin bound with higher affinity than DC-SIGN to gp140 with K_D2_ values of approximately 0.024 µM (BX08) and 0.011 µM (IIIB) (Fig. 1E and 1F).

### DC-SIGN, but not Langerin, increases the stability of gp140:CD4 complex

**Figure 2:**
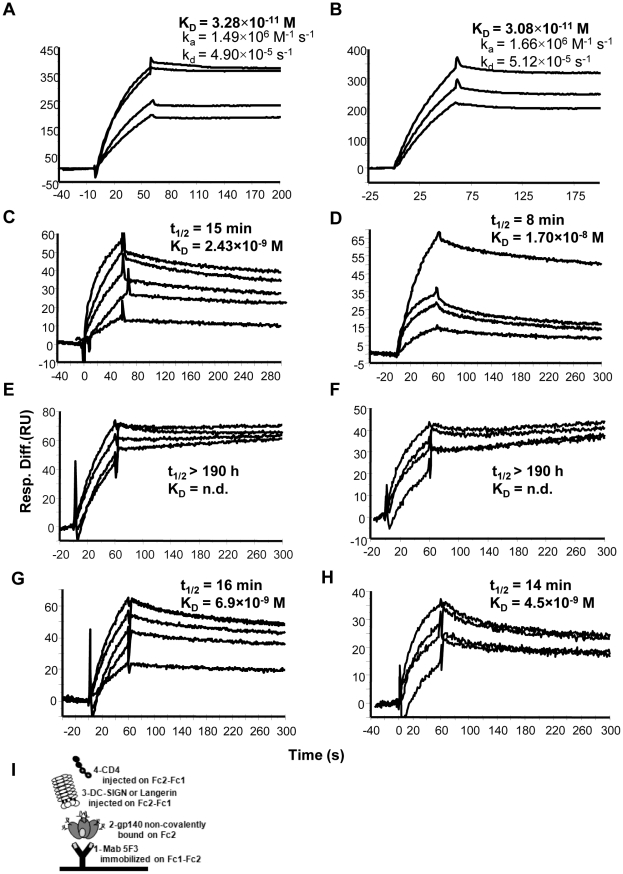
DC-SIGN, but not Langerin, stabilises the gp140:CD4 complex. (**A, B**) Superimposed sensorgrams of fluid phase gp140 BX08 (5–40 nM) or IIIB (5–10 nM), respectively, binding to immobilized Mab 5F3. Rate constants are indicated. (**C, E, G**) Superimposed sensorgrams of fluid phase CD4 at varying concentrations (50–500 nM) binding to gp140 (BX08) alone (**C**) and in complex with DC-SIGN (**E**) or Langerin (**G**). (**D, F, H**) Superimposed sensorgrams of fluid phase CD4 at varying concentrations (100–500 nM) binding to gp140 (IIIB) alone (**D**) and in complex with DC-SIGN (**F**) or Langerin (**H**). Sensorgrams shown were obtained by subtracting the background due to gp140:5F3 or DC-SIGN:gp140:5F3 or Langerin:gp140:5F3 dissociation from the curves obtained when CD4 was injected. Half-life values (t_1/2_ = ln 2/k_d_) are indicated. (**I**) Schematic representation of the surface plasmon resonance-based assay used. Gp140 was non-covalently bound on flow cell 2 to Mab 5F3 which was immobilized on flow cell 1 and flow cell 2 by direct amine coupling. Binding affinity of CD4 (injected on flow cell 1 and flow cell 2) was assessed in the presence or absence of DC-SIGN or Langerin bound to gp140.

To investigate whether formation of the complex of DC-SIGN with gp140 altered the affinity of interaction between gp140 and CD4 and to compare the effect of DC-SIGN with that of Langerin, a capture assay was used. The human anti-gp41 Mab 5F3 was immobilized on both experimental and reference flow cells followed by injection of gp140 over the surface of the experimental flow cell only. Mab 5F3 bound with high affinity to both preparations of gp140 with K_D_ values of approximately 3×10^−11^ M (Fig. 2A and 2B). As shown schematically in Fig. 2I, DC-SIGN or Langerin was then injected (over both flow cells) followed by CD4. Using this system, we compared CD4 binding to gp140 (BX08 and IIIB) complexed with DC-SIGN or Langerin to that of gp140 alone. Superimposed sensorgrams of fluid phase CD4 binding to gp140 (BX08) in the absence of DC-SIGN (with subtraction of gp140 dissociation from the capture antibody) are shown in Fig. 2C. Again, the data could not be fitted to the 1∶1 Langmuir binding model but were fitted to a two-step linked reaction binding model The apparent K_D_, 2.4 nM, is in agreement with previously reported values for CD4 interaction with gp120 from BH10 [51] and JRFL [52]. When DC-SIGN was injected and allowed to complex with gp140 (BX08) before injection of CD4, the kinetics of interaction were considerably altered. The superimposed sensorgrams (with subtraction of dissociation of the gp140:DC-SIGN:5F3 complex) (Fig. 2E), indicate a large decrease in the rate of dissociation of the complex. An accurate estimation of the dissociation rate was not possible and therefore other kinetic constants could not be calculated. We therefore calculated the half life (t_1/2_) of the respective gp140:CD4 complexes to compare their stability. For the gp140:CD4 complex formed in the absence of DC-SIGN, t_1/2_ was approximately 15 min whereas for the complex formed in the presence of DC-SIGN t_1/2_ was >190 h (assuming a k_d_ value of 10^−6^ s^−1^ as the limit of detection using surface plasmon resonance), consistent with an essentially irreversible interaction in this system. Although Langerin bound to gp140 (BX08) with higher affinity compared to DC-SIGN, no significant effect on CD4 binding was observed when gp140 was complexed with Langerin (Fig. 2G), with t_1/2_ of 16 min for the Langerin:gp140:CD4 complex. DC-SIGN also enhanced stability of the gp140 (IIIB) complex with CD4 (Fig. 2D and 2F). A small decrease in the dissociation rate of the gp140:CD4 complex was also evident in the presence of langerin (Fig. 2H). CD4 (without prior addition of gp140) did not bind to immobilized DC-SIGN or Langerin (data not shown).

### CD4-dependency of DC-SIGN enhancement of infection

To investigate whether the increased stability of the CD4 complex with gp140 contributes to infection with HIV, we compared the effects of *cis*-expression of DC-SIGN on *in vitro* infection with HIV-1 IIIB and HIV-1 IIIBx, a variant of IIIB that can bind directly to CXCR4 without CD4 [26], [37]. Any effect of enhancing infection by increasing the affinity of the envelope protein for CD4 should be reduced in a strain that does not have an absolute requirement for binding to CD4. This was tested by comparing infection of THP-1 cells, which express low levels of endogenous DC-SIGN, with infection of THP-1 cells transfected with DC-SIGN (THP-1DC-SIGN) where DC-SIGN is expressed at a higher level *in cis* on the cell surface membrane [24]. The DC-SIGN^+^ population identified within THP-1DC-SIGN and THP-1 by FACS analysis was 35% and <1% of total cells respectively (data not shown).

**Figure 3:**
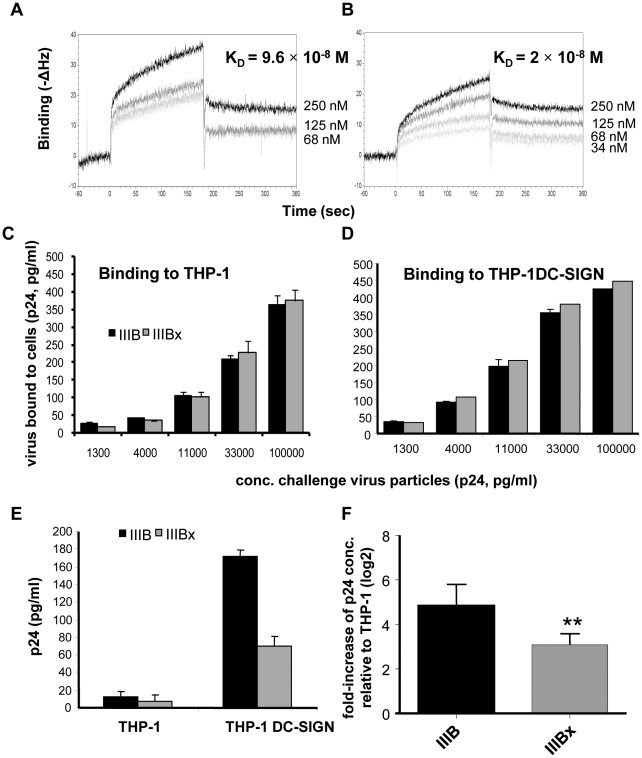
Binding properties and infectivity of CD4-dependent (IIIB) and CD4-independent (IIIBx) strains of HIV-1. (**A, B**) Superimposed sensorgrams (acoustic biosensor) of fluid phase IIIB (**A**) and IIIBx (**B**) viral particles (34–250 nM determined by p24 ELISA and normalized assuming 2500 gag proteins per virion) binding to immobilized DC-SIGN. K_D_ values are indicated. (**C, D**) Binding of IIIB and IIIBx to THP-1 (**C**) and THP-1 DC-SIGN (**D**) cells. Virus was incubated with cells for 2 h and after washing and cell lysis, p24 levels were estimated. Experiments were repeated twice, each point in triplicate. (**E**) Infection (10^−4^ MOI) by IIIB and IIIBx of THP-1 DC-SIGN and THP-1 cells represented as concentration of p24 antigen released in the supernatant. (**F**) Data from (**E**) as fold-increase in infection relative to THP-1. IIIBx infection level was significantly lower than IIIB (**, P = 0.001). Experiments were repeated twice (5 replicates).

Since the IIIBx strain lacks 5 N-linked glycosylation sites compared with IIIB [37], some of which may be bound by DC-SIGN [53], we first compared binding of IIIBx and IIIB virus particles to soluble DC-SIGN by using an acoustic biosensor system. IIIBx virus bound with higher affinity (K_D_ = 2.0×10^−8^ M, assuming 2,500 copies of p24/virus particle [46]) than IIIB (K_D_ = 9.6×10^−8^ M) (Fig. 3A and 3B). Binding of IIIBx and IIIB to THP-1 and THP-1DC-SIGN cells was also tested in p24 ELISA-based assays. In these experiments, binding levels to THP-1 and THP-1-DC-SIGN cells were similar for both strains at virus p24 concentrations of 1.3–100 ng/ml (Fig. 3C and 3D), but both strains bound 50–60% more to THP-1 DC-SIGN as compared to THP-1. These observations show that the CD4 independent strain IIIBx binds as well as IIIB to soluble DC-SIGN and THP-1DC-SIGN cells.

HIV-1 strains IIIB and IIIBx were then tested for infectivity in THP-1 and THP-1DC-SIGN cells. Significantly higher levels of p24 in the supernatants of THP-1DC-SIGN cells were detected than in the supernatants of THP-1 cells for both strains at 1–3×10^−4^ MOI with a higher level of enhancement evident at 10^−4^ MOI. At higher concentrations of virus, levels of p24 were similar in both cell types (data not shown). Data from a representative experiment where cells were infected with IIIB or IIIBx, both at 10^−4^ MOI, are shown in Fig. 3E and 3F. For both strains, higher levels of p24 were measured in THP-1DC-SIGN cells. However, whereas higher expression levels of DC-SIGN enhanced binding of IIIB and IIIBx to the same extent, the actual infection of the CD4 dependent IIIB is more enhanced (p = 0.001) than that of the CD4 independent IIIBx (34-fold increase for IIIB, 8-fold increase for IIIBx).

### Effect of *cis* expression of DC-SIGN on kinetics of cell fusion

**Figure 4:**
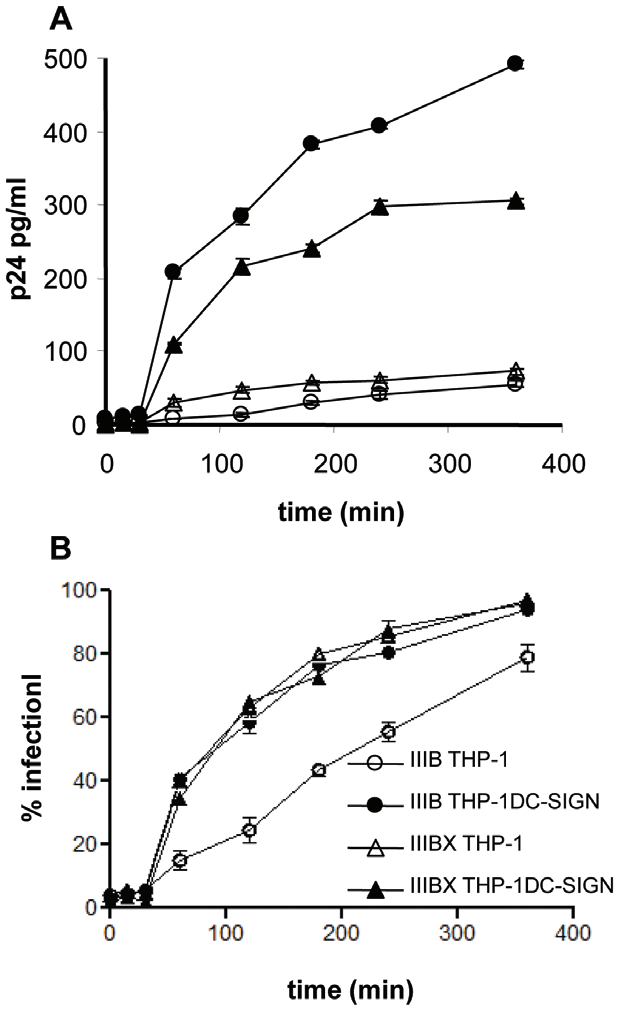
Effect of DC-SIGN on the kinetics of infection with IIIB and IIIBx. Virus was adsorbed to cells by spinoculation at 4°C. Cultures were warmed to 37°C and T-20 (completely inhibitory concentration) was added at the time points indicated. (**A**) Representative experiment showing levels of p24 measured in 4 day cultures following addition of T20. Each point was determined in triplicate. (**B**) Relative infection calculated as % p24 concentration relative to p24 concentration in 4 day cultures with no T-20. Levels of p24 in the absence of T-20 were determined in triplicate for each strain in each cell type in 4 separate experiments. Error bars are standard errors of means (n = 4).

Using the same system, we then compared the effect of DC-SIGN on the kinetics of infection with IIIB and IIIBx strains. For these experiments, HIV was adsorbed to cells by spinoculation at low temperature to promote synchronous infection [48], [49]. Cultures were warmed to 37°C and the fusion inhibitor T-20 was added to cultures at defined time intervals and at a completely inhibitory concentration. The rate at which virus became resistant to T-20 inhibition provides a measure of the rate of completion of HIV fusion. There was no detectable infection when T-20 was added to cultures up to 30 minutes after warming (Fig. 4A) indicating the time required in this system for formation of the fusion complex and subsequent adoption of the helix bundle conformation. At the next time point (60 minutes) infection of both THP-1 and THP-1DC-SIGN cells with both IIIB and IIIBx strains was evident. As before, levels of infection were higher in THP-1DC-SIGN cells (Fig. 4A). A plot of relative infection against time (Fig. 4B) revealed significant differences in the rate of infection of the two strains of HIV. Despite the difference in level of infection (Fig. 4A), the relative rate of infection by HIV IIIBx was similar for both THP-1 and THP-1DC-SIGN cells whereas that of infection by IIIB was slower in THP-1 cells compared with THP-1DC-SIGN cells (Fig. 4B). The more efficient capture of virus by DC-SIGN leads to higher levels of infection but, in addition, for a wholly CD4-dependent strain the relative rate of infection is increased. We suggest that the increased rate is a consequence of DC-SIGN stabilising the gp120:CD4 complex. Addition of Mab b12 [28] at any time after spinoculation had no effect, consistent with the finding that virus binds to CD4 during spinoculation [48].

### Effect of soluble DC-SIGN on HIV-1 infection *in vitro*

**Figure 5:**
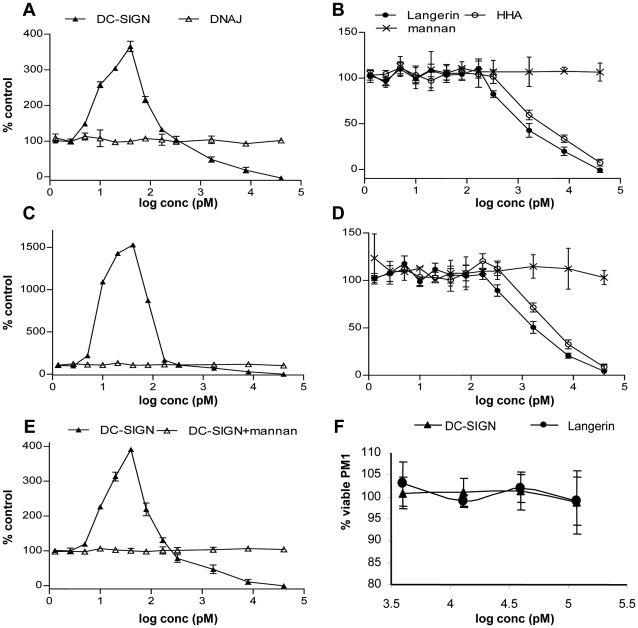
DC-SIGN, but not Langerin, enhances replication in PM1 cells by HIV-1. Levels of p24 are represented as %p24 production in the control wells, which was defined as 100%. Each condition was assayed in triplicate in two independent experiments. (**A, B**) Effect of soluble DC-SIGN (**A**), Langerin and HHA (**B**) on PM1 cells infection by HIV-1 (BX08). (**C, D**) Effect of soluble DC-SIGN (**C**), Langerin and HHA (**D**) on PM1 infection by HIV-1 (IIIB). DnaJ (▵), used as control protein, and mannan alone (×) showed no significant effect. (**E**) Effect of soluble DC-SIGN at increasing concentrations on PM1 cells infection by HIV-1 (BX08) in the presence (▵) and absence (▴) of mannan at 10 µg/ml. (**F**) Effect of DC-SIGN and Langerin on viability of PM1 cells assessed by MTT dye reduction assays.

The data reported above indicate that *cis* expression of DC-SIGN enhances infection with HIV-1 both by concentrating virus at the cell surface and by stabilising binding of gp120 to CD4. We compared the effects of soluble DC-SIGN or soluble Langerin on HIV infection *in vitro* as a means of addressing whether the increased stability of the CD4:gp120 complex contributes to DC-SIGN enhancement of *trans* infection. In a previous study (28), inhibition of infection of T cells was evident when soluble DC-SIGN was added to virus *in vitro* at concentrations in the range of 2–200 nM. Furthermore, in separate assays, virus capture was reduced by approximately 50% following addition of soluble DC-SIGN at a concentration of approximately 2 nM. We therefore carried out a titration of DC-SIGN that included sub-nanomolar concentrations. DC-SIGN, Langerin or the plant lectin HHA was added to virus particles prior to addition of PM1 cells and p24 levels were determined after culture for 10 days. HHA has previously been identified as a microbicide that binds to HIV gp120 [54] [55]. At concentrations of DC-SIGN <1 nM, significantly increased p24 levels were evident in the supernatants of both BX08 and IIIB strains compared to the control (no treatment) with maximum enhancement of 3.6-fold and 15.3-fold, respectively (Fig. 5A and 5C). At higher concentrations, DC-SIGN inhibited replication, as previously described (28), with IC_50_ of 2.5 nM (BX08) and 4 nM (IIIB). In contrast, Langerin, and HHA had no enhancing effect at sub-nanomolar concentrations but inhibited infection with both HIV-1 strains in a dose-dependent manner with IC_50_ values in the range of 1.6–5 nM (Fig. 5B and 5D), as previously reported for HHA (1). Both enhancing and inhibitory activities of DC-SIGN were abrogated in the presence of mannan (Fig. 5E) confirming that the effects are mediated by carbohydrate binding. DnaJ (included as a control recombinant polypeptide of irrelevant specificity) showed no significant effect. None of the compounds showed toxicity in the MTT dye reduction assay (Fig. 5F).

Both (enhancing) DC-SIGN and (non-enhancing) HHA lectins bound to PM1 cells as observed by FACS analyses (data not shown). At the highest concentration tested (300 nM) for each lectin, the level of binding determined as mean fluorescence intensity (MFI) of HHA to PM1 cells was higher (MFI = 22.15, 89.4%) than that observed for DC-SIGN (MFI = 8.89, 14.7%). We cannot therefore exclude the possibility that enhancement of infection was due to tetravalent DC-SIGN acting as a bridge to bind virus to the PM1 cell surface. However, the observation that tetravalent HHA binds more strongly to PM1 cells suggests that bridging *per se* is not sufficient to enhance infection. We suggest that at low concentrations, soluble DC-SIGN but not Langerin (or HHA) stabilises interaction of gp120 on the virus surface with CD4 to enhance infection. At higher concentrations, binding of more than one molecule of soluble DC-SIGN to gp120 [53] may sterically hinder binding to cell surface DC-SIGN.

### DC-SIGN increases binding affinity of gp140 to anti-CD4-binding site neutralizing Mab b12, but not to an anti-V3 Mab

**Figure 6:**
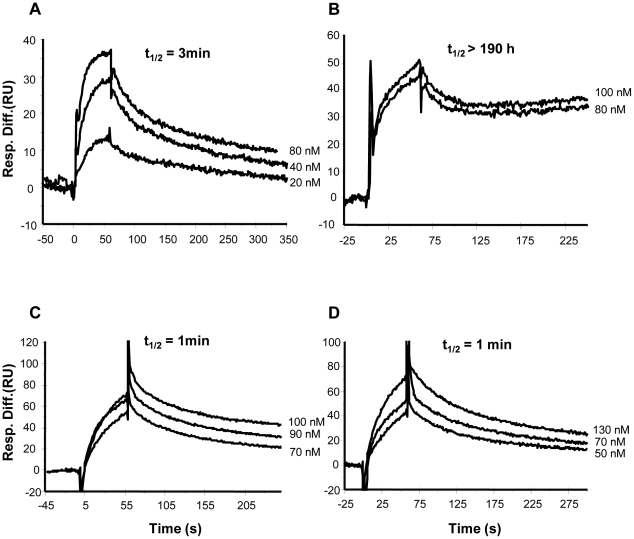
DC-SIGN increases binding affinity of gp140 to Mab b12, but not to Mab 447-52D. (**A, C**) Superimposed sensorgrams representing binding of fluid phase Mab b12 (**A**) and Mab 447-52D (**C**) at the concentrations indicated to gp140 (BX08) alone. (**B, D**) Superimposed sensorgrams representing binding activity of fluid phase Mab b12 (**B**) and Mab 447-52D (**D**) at the concentrations indicated to gp140 (BX08) in complex with DC-SIGN. Gp140 was non-covalently bound on flow cell 2 to Mab 5F3 which was immobilized on flow cell 1 and flow cell 2 by direct amine coupling. Binding affinity of the antibodies (injected on flow cell 1 and flow cell 2) was assessed in the presence or absence of DC-SIGN also injected over both flow cells.

The enhanced binding of CD4 to gp140 induced by DC-SIGN may result from increased availability of the CD4 binding site. We tested the effect of DC-SIGN on gp140 interaction with Mab b12 directed against the CD4 binding site and of Mab 447-52D directed against the V3 loop of gp120 [29]. As described above, gp140 was non-covalently bound on the experimental flow cell by capture using immobilised Mab 5F3. Binding of Mabs b12 or 447-52D at various concentrations was then measured in the presence or absence of DC-SIGN. Fig. 6A shows superimposed sensorgrams of fluid phase Mab b12 binding to gp140 (BX08) alone. The data fitted the two-step dissociation model of binding with apparent K_D_ 7.9×10^−9^ M in good agreement with previous findings [56]. When DC-SIGN was bound to gp140 by prior injection, dissociation of Mab b12 from the DC-SIGN:gp140 complex was considerably reduced (Fig. 6B). The rate of dissociation (and therefore kinetic constants) could not be determined (as shown in Fig. 2 for CD4 binding to the same complex). Binding of Mab 447-52D to gp140 in the presence or absence of DC-SIGN was also measured in this system. DC-SIGN had no significant effect on interaction of Mab 447-52D with gp140 (Fig. 6C–D).

## Discussion

In this study, we have demonstrated that DC-SIGN-mediated enhancement of infection with HIV-1 is the result not only of increasing the concentration of virus at the cell surface as suggested previously [24] but also of the increased affinity of the DC-SIGN:gp120 complex for CD4. The C-type lectins DC-SIGN and Langerin both bind to HIV-1 envelope protein but with different outcomes. Whereas binding of HIV to DC-SIGN may enhance infection, binding of HIV to Langerin leads to internalisation and degradation of HIV, as outlined above. To compare directly the effect of DC-SIGN and Langerin binding to HIV-1 envelope protein, we developed a surface plasmon resonance-based binding assay in which gp140 was immobilised indirectly on the sensorchip surface by antibody, and binding of soluble CD4 could be reproducibly measured with or without prior binding of soluble DC-SIGN or soluble Langerin. The findings revealed qualitative differences between binding of the two C-type lectins. In this system, binding of DC-SIGN to immobilised gp140 resulted in a large increase in the affinity with which the complex bound to CD4 compared with binding of gp140 alone. The increase in affinity was mainly due to a decrease in the dissociation rate of the trimolecular DC-SIGN:gp140:CD4 complex compared with gp140:CD4. In contrast, Langerin had little or no effect on the dissociation of CD4 from the complex with gp140. These findings confirm those reported previously where DC-SIGN binding to gp120 was shown to enhance binding of the complex to CD4 using an ELISA-based system [57].

For the binding studies reported here, gp140 produced in human 293T cells was used so that the glycosylation pattern more closely resembles that of native envelope proteins of HIV-1 particles. Previous studies of HIV-1 gp120 produced in insect or mammalian cells demonstrated marked differences in binding to CD4 and some antibodies of the differently glycosylated forms [58]. Significant differences in glycosylation between gp120 produced in 293T cells or in a T cell line (Jurkat) have also been described [59] with 293T cell-derived material having less high mannose and more complex N-linked glycans than the Jurkat-derived material. However, in contrast to gp120, gp140 produced in 293T cells shows a much simpler, predominantly oligomannose profile of glycans that correlates well with the glycan profiles of envelope proteins purified from functional virus particles produced in 293T cells or human blood peripheral mononuclear cells [60]. We also used preparations of DC-SIGN and Langerin that were mostly tetrameric or trimeric, respectively, reflecting the oligomeric structure of the cell surface forms of these lectins [41], [61] and in which binding was predominantly by the oligomeric forms.

Although both DC-SIGN and Langerin bind to oligomannose structures on the HIV envelope protein, significant differences in the binding properties of DC-SIGN and Langerin revealed by crystallographic studies [62], [63] may account for the observation that DC-SIGN but not Langerin increases the affinity of gp140 for CD4. DC-SIGN binds preferentially to the outer trimannose branch point within N-linked high mannose glycans [62] whereas Langerin binds preferentially to linear oligomannoses [64], [65]. The carbohydrate recognition domain of Langerin includes a novel second carbohydrate binding site in addition to the site conserved in C-type lectins [66]. Modelling studies suggest that N-linked high mannose glycans may bind to Langerin through a terminal mannose residue of one branch at the conserved site and two mannose residues of another branch at the alternative site [67]. In addition, the 3 carbohydrate recognition domains within the Langerin trimer are fixed in position by multiple interactions with the neck region [64] and likely to bind to a preformed target site. In contrast, the carbohydrate recognition domains of tetrameric DC-SIGN are more flexible [63] and may therefore bind to a wider range of glycans.

To determine whether the increased affinity of the DC-SIGN:envelope bimolecular complex binding to CD4 contributed to DC-SIGN mediated *cis* enhancement of infection, as suggested previously [57], we used a cellular model of infection [18], [24] that compares infection of DC-SIGN transfected and non-transfected THP-1 cells. Although the effect of DC-SIGN was to increase infection by both CD4-dependent and CD4-independent strains of HIV, the level of enhancement was consistently lower for the CD4-independent strain. In the same model, we also determined kinetics of infection for both HIV strains in the two cell types. For this we used spinoculation at low temperature to produce synchronous infection of cells [48], [49] and then measured the rate at which inoculated HIV became resistant to the fusion inhibitor T-20, corresponding to the time required for adoption of the six-helix bundle conformation of gp41 [68]. In contrast to IIIBx where the relative rate of infection is not affected by DC-SIGN, the relative rate of infection of IIIB was increased by *cis* expression of DC-SIGN such that it was similar to that of the CD4-independent strain. Thus the increased stability of gp120:CD4 interaction conferred by DC-SIGN contributes in turn to faster formation of the six-helix bundle. Binding analyses combined with structural determination [69] have indicated rapidly reversible binding of CD4 to gp120 in which CD4 binds to a site that is constitutively exposed but then readily dissociates. The complex may be stabilised by multiple copies of CD4 at the surface of T cells. In contrast, the low levels of cell surface CD4 in DCs [11] may not provide the increased avidity required to stabilise the initial gp120:CD4 complexes. Data from this study shows that DC-SIGN may significantly contribute to *cis* infection by decreasing the dissociation of CD4, thereby promoting formation of the complex with gp120 leading to more rapid co-receptor binding and fusion. This may be a significant factor in infection of DCs. The IIIBx strain retains CD4 binding activity showing enhanced fusion activity when CD4 and CXCR4 were coexpressed on target cells for fusion assays [37] and we cannot rule out such activity contributing to infection in the assays performed in this study. The observation that the relative rate of infection with IIIBx is not affected by *cis* expression of DC-SIGN, however, suggests that DC-SIGN enhances infection of this strain only by increasing the level of virus binding to the cell surface. It is striking that DC-SIGN increases the relative rate of infection with the parental IIIB strain so that it resembles that of IIIBx indicating that the increased stability of the DC-SIGN:Env:CD4 complex may lead to more rapid co-receptor engagement.

The system used here of comparing CD4-dependent and CD4-independent virus strains in the presence or absence of DC-SIGN to distinguish between the DC-SIGN-mediated effects of increasing binding of virus to the cell surface and increasing the stability of the gp120:CD4 complex cannot be applied to *trans* enhancement of infection. We therefore investigated the effect of inoculating virus in the presence of soluble DC-SIGN in comparison with the effect of adding soluble Langerin or HHA. At low concentrations, only DC-SIGN mediated enhancement of infection whereas at higher concentrations all lectins were inhibitory. We suggest that inhibition is due to excess bound lectin sterically blocking the site for binding CD4 whereas, at low concentrations of DC-SIGN, DC-SIGN:envelope complexes on the virus surface bind more avidly to CD4 on the target cells. The multivalent lectins used in this experiment recognise carbohydrate structures on the host cell and could enhance infection by acting as a bridge to target HIV to the cell surface. We demonstrated, however, that although both HHA and DC-SIGN bound to the PM1 cells, used as permissive targets, only DC-SIGN enhanced infection suggesting that the specificity of lectin interaction but not bridging contributes to enhancement.

We also showed that DC-SIGN increases the affinity of binding of the broadly neutralising monoclonal antibody b12 which recognises an epitope that largely overlaps with the CD4 binding site of gp120 [69] consistent with the proposal that DC-SIGN exposes the CD4 binding site. Similarly, binding of the mannose-rich glycan-specific lectin, griffithsin, to gp120 has been shown to enhance binding of b12 and modestly enhances binding of a soluble form of CD4 [70]. The N-linked glycan at position 386 contributed partly to griffithsin-mediated enhancement of binding. This glycan was also identified as a component of the optimal DC-SIGN binding site on gp120 [53]. Molecular modelling suggests that this glycan shields the CD4 binding site and enhanced macrophage tropism was observed in a HIV Env variant that lacked the glycan at position 386 [71]. Thus we suggest that DC-SIGN binding to the N-linked glycan at position 386 may contribute to exposure of the CD4 binding-site and DC-SIGN-mediated enhancement of infection. This glycan is not essential for DC-SIGN-mediated virus capture since it is one of five N-linked glycans lacking in gp120 of the IIIBx strain [37].

Binding of HIV to DC-SIGN on the surface of DCs may have a number of different outcomes. In the absence of productive infection, virus may be transmitted *in trans* to permissive target cells. In addition, by concentrating virus at the DC cell surface, DC-SIGN increases the likelihood of interaction *in cis* with DC cell surface CD4 and co-receptor as suggested previously [22], [24] leading to infection *in cis*. In parallel, DC-SIGN also mediates internalisation and degradation of HIV in endosomes [16], [17]. Recognition of released HIV-1 genomic RNA by TLR8 and binding of HIV-1 envelope protein to DC-SIGN provide two signals that are essential for initiation of transcription and production of full length viral transcripts [72], thus also enhancing infection *in cis*. In this study, we demonstrate a further effect of DC-SIGN that contributes to enhanced infection either *in cis* or *in trans*, namely significantly increasing the half-life of the envelope glycoprotein:CD4 complex which in turn promotes co-receptor engagement.
